# Interrelated atrial fibrillation and leaks triggering and maintaining central sleep apnoea and periodic breathing in a CPAP‐treated patient

**DOI:** 10.1002/rcr2.666

**Published:** 2020-09-22

**Authors:** Arnaud Prigent, Anne‐Laure Serandour, Régis Luraine, Jean Sébastien Poineuf, Christian Bosseau, Jean‐Louis Pépin

**Affiliations:** ^1^ Groupe Médical de Pneumologie Polyclinique Saint‐Laurent Rennes France; ^2^ Centre du sommeil Polyclinique Saint‐Laurent Rennes France; ^3^ SLB Pharma Rennes France; ^4^ Service de cardiologie Polyclinique Saint Laurent Rennes France; ^5^ HP2 Laboratory, Inserm Unit 1042 University Grenoble Alpes Grenoble France

**Keywords:** Atrial fibrillation, central sleep apnoea, CPAP leaks, periodic breathing, trigger

## Abstract

We report the case of a 71‐year‐old obese continuous positive airway pressure (CPAP)‐treated man who developed an acute cardiac failure (ACF) triggered by atrial fibrillation. CPAP data downloaded from the CPAP software (Rescan®) retrospectively demonstrated the progressive development of a high residual central apnoea–hypopnoea index (AHI) with Cheyne–Stokes respiration (CSR). The AHI decreased after cardioversion allowing normalization of cardiac rhythm and function. Raw data extracted from CPAP software showed a gradual decrease in the periodic breathing cycle length related to a simultaneous improvement in left ventricular ejection fraction (LVEF) after cardioversion. During this clinical period of respiratory instability in the presence of cardiac failure, CSR episodes were exacerbated by ventilation overshoots followed by micro‐arousals induced by leaks. This might explain the high night to night variability of CSR occurrence in susceptible patients with impaired cardiac function. Beyond attempts to improve cardiac function, leak reduction might represent an important target for CSR management.

## Graphical abstract

Prior to acute cardiac failure triggered by atrial fibrillation, data from the patient's continuous positive airway pressure (CPAP) device demonstrated the progressive development of high residual central apnoea–hypopnoea index with Cheyne–Stokes respiration (CSR) that both decreased after cardioversion with normalization of cardiac rhythm and function, decrease in periodic breathing cycle length, and improvement in left ventricular ejection fraction (LVEF). CSR episodes were exacerbated by ventilation overshoots following micro‐arousals induced by leaks.

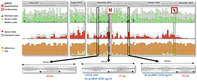

## Introduction

In individuals treated for obstructive sleep apnoea (OSA) syndrome, data downloaded from continuous positive airway pressure (CPAP) devices can detect changes in the residual apnoea–hypopnoea index (AHI) and periodic breathing patterns. Periodic breathing and its cycle length have been demonstrated to be linked to cardiac output [1].

## Case Report

A 71‐year‐old obese (body mass index (BMI) = 34 kg/m^2^) man was admitted for acute cardiac failure (ACF) triggered by atrial fibrillation (heart rate: 168 beats per minute, N‐terminal prohormone of brain natriuretic peptide (NT‐pro‐BNP): 6050 pg/mL, left ventricular ejection fraction (LVEF): 35%). He has been CPAP‐treated for 10 years for severe OSA and demonstrated high CPAP adherence (average usage: >8 h/night, effective pressure: 8 cm H_2_O) and normalization with a residual AHI below 5/h. His medical history included asthma and psoriasis, but no overt cardiovascular disease. In the days preceding admission, he reported perceived extra systoles and arrhythmia episodes, dyspnoea, and reported apnoeas.

Diuretics and other recommended medications targeting improvement in cardiac function (beta‐blocker and angiotensin‐converting enzyme) in association with oral anticoagulants were prescribed before an electrical cardioversion scheduled one month later. The cardioversion allowed conversion to a sinus rhythm. His NT‐pro‐BNP gradually decreased and normalized in two months. Meanwhile, echocardiography proved a complete LVEF (63%) recovery in a patient with no persistent symptoms of heart failure. This LVEF normalization following the correction of chronic tachycardia (atrial fibrillation in this case) confirmed the diagnosis of tachycardia‐induced cardiomyopathy, also called tachycardiomyopathy.

**Figure 1:**
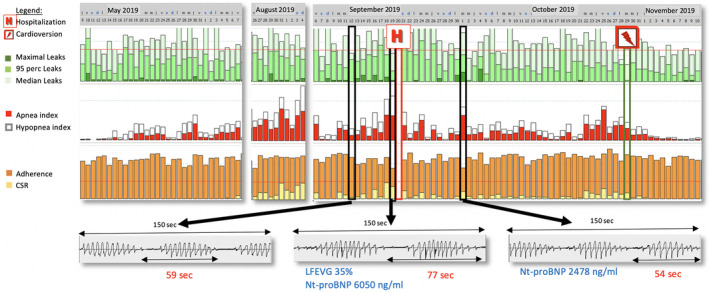
Report from the downloaded continuous positive airway pressure (CPAP) software from 9 May to 10 November 2019.

CPAP data downloaded (Fig. 1) from the CPAP software (Rescan®, Resmed, Australia) retrospectively demonstrated the progressive appearance of a high residual AHI with Cheyne–Stokes respiration (CSR) in parallel with an atrial fibrillation episode starting during the weeks preceding hospitalization for ACF. AHI decreased after cardioversion and normalization of cardiac function. Leak‐related micro‐arousals were aggravated by the occurrence and maintenance of central events and CSR.

## Discussion

**Figure 2:**
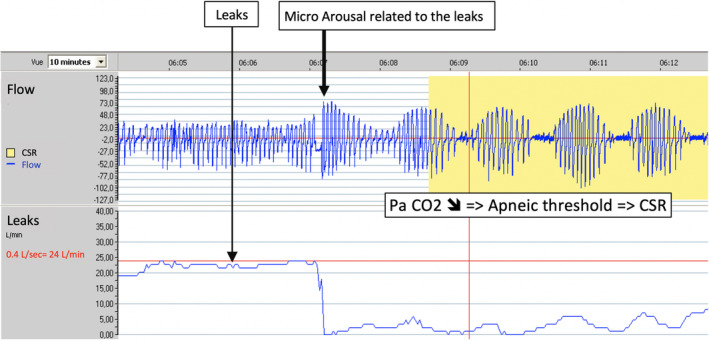
High‐resolution data downloaded from continuous positive airway pressure (CPAP) recordings (large leaks/micro‐arousals).

This case is a nice clinical demonstration of the interplay between periodic breathing and its cycle length (Fig. 1), and cardiac output. A novelty of our case report is to decipher two distinct and synergistic CSR‐triggering mechanisms. In the context of cardiac failure, the main driver of CSR is related to ventilation instability with chronic hyperventilation and low partial pressure of carbon dioxide (PaCO_2_) in the arterial blood. In this context, the occurrence of a micro‐arousal of any cause generates a ventilatory overshoot (“plant gain”) visualized by the crescendo phase of CSR and the decrease in PaCO_2_ crossing the apnoeic threshold and thus reducing ventilation producing central hypopnoeas and apnoeas (Fig. 2) [1]. Any underlying cause of sleep fragmentation, mainly leaks in CPAP‐treated patients but also residual obstructive events, acts as a starting point for a CSR vicious circle (Fig. 2). It is important to acknowledge that the monitoring system does not record some other critical data that might influence the emergence or worsening of CSR (e.g. body position or medication administration such as opioids).

There is a clear association between the severity of heart failure whatever the underlying mechanisms and CSR [1]. The capability of CPAP telemonitoring for the early detection of cardiovascular events (heart failure [2] or atrial fibrillation [3]) has already been suggested by case reports but this should now be prospectively documented by additional studies conducted in larger real‐life CPAP‐treated populations (see ongoing trial: NCT03592108). The understanding of mechanisms triggering and perpetuating CSR under CPAP is extended by this case report [4, 5]. The respective roles of micro‐arousals induced by leaks or by residual obstructive events are reported for the first time using CPAP data downloads. This knowledge might explain the high night to night variability of CSR occurrence in susceptible patients. This better understanding should now translate into appropriate clinical management by addressing more cautiously leaks in complement to state‐of‐the‐art cardiac treatment.

### Disclosure Statements

Appropriate written informed consent was obtained for publication of this case report and accompanying images.

A. Prigent is a consultant for Resmed. J.‐L. Pepin has received grants and personal fees from Resmed and from Philips, and grants from Fisher and Paykel, all outside the context of the present work. A.‐L. Serandour, R. Luraine, J. S. Poineuf, and C. Bosseau do not have any conflict of interest or potential conflict of interest to declare.
